# *TP53* p.R181H is enriched in the Swedish cohort (SWEP53) and associated with a distinct breast and prostate phenotype

**DOI:** 10.1038/s41598-025-22407-2

**Published:** 2025-10-07

**Authors:** Alexander Sun Zhang, Meis Omran, Cecilia Arthur, Helena Malmgren, Rita Barbosa-Matos, Susana Seixas, Carla Oliveira, Emma Tham, Svetlana Bajalica-Lagercrantz

**Affiliations:** 1 Department of Oncology-Pathology, Karolinska Institutet, J6:20 Bioclinicum, Akademiska Stråket 1, 171 77 Stockholm, Sweden; 2 Cancer Theme, Karolinska University Hospital, Stockholm, Sweden; 3 Department of Molecular Medicine and Surgery, Karolinska Institutet, Stockholm, Sweden; 4 Department of Clinical Genetics and Genomics, Karolinska University Hospital, Stockholm, Sweden; 5 Instituto de Investigação E Inovação Em Saúde, University of Porto (i3S), Porto, Portugal; 6 Institute of Molecular Pathology and Immunology of the University of Porto (IPATIMUP), Porto, Portugal

**Keywords:** Germline *TP53*, Li-Fraumeni syndrome, Founder, Surveillance, Cancer, Genetics, Oncology

## Abstract

Li-Fraumeni Syndrome (LFS) is a severe cancer predisposition syndrome caused by germline *TP53* variants and characterized by a wide range of cancers. The *TP53* variant p.R181H is enriched in the Swedish germline *TP53* cohort and has distinct phenotypical characteristics. Using our nationwide Swedish germline *TP53* database (SWEP53, 189 individuals, 86 families), p.R181H was identified in 22% of families (19/86), compared to just 0.6% in the National Cancer Institute (NCI) *TP53* database (8/1360). Notably, p.R181H carriers had lower cancer incidence and better survival than carriers of other *TP53* variants (both p < 0.0001). Female carriers primarily developed breast cancer (earliest onset 29 years), males mainly prostate cancer (earliest onset 45 years), and no cancers were observed in children. The results were confirmed using the NCI *TP53* database. Tumor sequencing confirmed loss of heterozygosity. Additionally, haplotype analysis suggests that p.R181H is a potential Swedish founder variant, estimated to be ~ 550 years old. These findings indicate that, for p.R181H carriers, tailored surveillance could focus on adults by omitting children from testing and surveillance, and by targeting prevention and detection to breast cancer in females and prostate cancer in males. Validation in independent cohorts is warranted.

**Supplementary Information:**

The online version contains supplementary material available at 10.1038/s41598-025-22407-2.

## Introduction

Li-Fraumeni Syndrome (LFS), also known as heritable *TP53*-related cancer syndrome (h*TP53*rc), is a hereditary cancer predisposition syndrome caused by germline variants in the tumor suppressor gene *TP53*^1,2^. The syndrome is inherited in an autosomal dominant manner and the lifetime cancer risk is approximately 70% for males and close to 100% for females^3,4^. Moreover, approximately 50% of affected individuals who develop an initial malignancy will develop a subsequent primary cancer^5^. Adrenocortical carcinomas (ACC), breast cancers, central nervous system (CNS) tumors, osteosarcomas and soft-tissue sarcomas represent the most frequently observed malignancies and are therefore considered the core cancers of LFS. However, the cancer spectrum is broad and includes a variety of other malignancies, such as leukemia, lung cancer, and skin cancer^6,7^. LFS patients also display a heterogenous temporal spectrum with certain variants associated with earlier onset of first cancer^8^. Due to the extreme lifetime cancer risk, current international guidelines recommend intensive tumor screening programs including whole-body magnetic resonance imaging (WB-MRI), abdominal ultrasounds and clinical check-ups. Unfortunately, these screening programs are often one-size-fits-all and fail to take into consideration the various cancer spectra and temporal distribution exhibited by different *TP53* variants^1,9^.

There is a wide spectrum of *TP53* variants that are known to cause LFS, and cohorts usually show a large variant and phenotype heterogeneity. To date, more than 1,500 unique *TP53* germline variants have been reported in the National Cancer Institute (NCI) *TP53* database^10^. In certain populations specific germline variants can be enriched and observed in a higher frequency than expected. These variants are known as founder variants and originate from a common ancestor in a specific geographical region. Founder variants share the same haplotype, which distinguishes them from hot-spot variants which are identified on different haplotype backgrounds^10–14^. Only a few founder variants in *TP53* have been described^15–17^. The most studied founder variant, p.R337H, is prevalent in parts of Brazil and is thought to have originated from Portuguese migrants^18,19^. While p.R337H exhibits lower overall cancer penetrance, carriers have higher incidence of ACC, papillary thyroid cancer, renal cancers and lung adenocarcinomas compared to other *TP53* variants. Similarly, the other founder variants in *TP53* also exhibit a wide cancer spectrum necessitating the use of WB-MRI for tumor surveillance. Consequently, there are no founder variants with enough clinical homogeneity to enable personalized clinical management^12,16,17,20–22^.

Here, we used our Swedish germline *TP53* database to identify p.R181H (NM_000546.6:c.542G>A) as a highly enriched variant in the Swedish population. Carriers of the variant exhibited a clinically distinct phenotype characterized by absence of childhood cancers, low cancer penetrance and significantly improved survival. Additionally, the cancer spectrum was narrow and dominated by breast and prostate cancer. The homogenous phenotype could therefore be exploited to enable personalized clinical management.

## Methods

### Swedish germline *TP53* database (SWEP53)

Variant data and clinical data on Swedish LFS families were retrieved from the Swedish germline *TP53* database (SWEP53, last update August 2024, 189 individuals from 86 families)^23^. The nationwide database includes all known *TP53* carriers in Sweden with a likely pathogenic (class 4) or pathogenic (class 5) *TP53* variant (Table S1)^24,25^. Patients with multiple variants are not included. Used variables from the database included variant data, pedigree, age at cancer onset, cancer type, pathological reports and survival data. All families were then classified into one of the phenotypical groups: classic LFS, Chompret or hereditary breast cancer (HBC). Families that could not be classified into either of the three phenotypical groups were classified as “other” (Table S2).

### Publicly available datasets

The *TP53* database (version R20, January 2024) hosted by NCI aggregates germline *TP53* variant carriers from published literature since 1989. Data includes age at cancer onset, cancer type and survival information^10^. Data was retrieved in February 2024. Only individuals with confirmed or mandatory *TP*53 carriership that were classified as likely pathogenic (class 4) or pathogenic (class 5) were included, all patients with multiple *TP53* variants were excluded. In total 1,995 individuals (1,360 families) were selected for further downstream analysis. All patients not classified as either classic LFS or Chompret were classified as “other” since the HBC phenotypical group is not used in the database. For analyses of cancer incidence using the NCI *TP53* database, p.R181H carriers from the Swedish cohort were pooled with those from the NCI *TP53* cohort to increase statistical power, as the number of p.R181H carriers in the NCI dataset alone was insufficient for robust analysis. The comparator group consisted of all other variant carriers within the NCI *TP53* cohort. This allowed external confirmation of findings while avoiding cohort mixing in the comparator group. No survival data were available for p.R181H carriers in the NCI *TP53* cohort; therefore, survival analyses included only Swedish p.R181H carriers, with the comparator group drawn from the NCI *TP53* cohort. Additionally, we used variant frequency data from the NCI longitudinal Li-Fraumeni syndrome study which included a total of 480 carriers of likely pathogenic or pathogenic *TP53* variants^26^.

For healthy (non-cancer) population allele frequencies, we used the Genome Aggregation Database (gnomad.broadinstitute.org version 2.1.1, non-cancer). Additionally, we used the FLOSSIES database (Fabulous Ladies Over Seventy) which contains data from targeted sequencing of 27 genes associated with breast cancer including *TP53.* In total 10,000 females, over the age of 70 years, who have never had cancer were included (whi.color.com).

### Haplotyping and age estimation

Twelve short tandem repeat (STR) markers flanking the *TP53* gene were analyzed (Table S3). All p.R181H variant carriers in the Swedish cohort with available leukocyte genomic DNA were analyzed (18 individuals from seven families). For all families, except family E (Table S3), samples from at least two generations were available, making it possible to better determine the haplotypes and sort the alleles. Primers for the STR markers were retrieved from the University of California, Santa Cruz (UCSC) database and analyzed by conventional PCR and fragment length analysis (ABI3500XL, Thermo Fisher Scientific). The EstiAge software was used to estimate the age of the most recent common ancestor (TMRCA) based on the genetic distance of haplotypes sharing between independent families, assuming a 0.001 global mutation rate per meiosis. Each generation was assumed to be 25 years^27^.

### AlphaMissense

The deleteriousness of the variant was computationally assessed using AlphaMissense, a deep learning model trained to predict pathogenicity based on protein sequence input^28^. The model was accessed through the web interface developed by Tordai et al. with variants classified into three categories according to their pathogenicity scores: benign/likely benign (0–0.34), uncertain significance (0.34–0.564), and likely pathogenic/pathogenic (0.564–1)^29^.

### Tumor sequencing

DNA was purified from formalin-fixed paraffin-embedded (FFPE) tumor tissue and sequenced on a NovaSeq 6000 (Illumina) using a paired-end 150 nucleotide readout, aiming at 40 million read pairs per sample using a custom-designed hybridization capture gene panel (GMCKv1, Twist Bioscience) with 387 genes^30^. Data was analyzed using the Balsamic pipeline version 5.1.0. (https://balsamic.readthedocs.io/en/latest/) and visualized in SCOUT. Variants were manually checked using quality values (total depth > 100; variant depth > 10; variant allele frequency > 3%) and variant databases (gnomAD v2.1.1), Clinvar, COSMIC and our internal database.

### Statistical analysis and visualization

Independent t-test was used for comparisons between two continuous variables when normally distributed. Chi-squared test was used for comparisons across two or more categorical variables if the expected value in each cell was higher than five, otherwise Fisher’s exact test was used. Overall survival and cumulative incidence were estimated using the Kaplan–Meier method and the log-rank test was used to calculate the p-values. All individuals, including those who did not develop cancer during follow-up, were included in the survival and cumulative incidence analyses. In cases where p.R181H carriers would otherwise have been included in a comparator group (e.g., HBC or missense groups), they were removed from that group to avoid overlap. Statistical significance was set at *p* < 0.05. All statistical computing was performed using the R programming language (version 4.2.2) in RStudio (version 2023.06). The package “dplyr” was used for data wrangling, “ggplot2” was used for creating figures and “survminer”, “survival” and “ggsurvfit” were used for survival analysis, Kaplan–Meier curves and cumulative incidence curves. Adobe Illustrator (version 28.6) was used for further figure annotation and layout adjustment.

### Ethical considerations

This study was performed in accordance with the Declaration of Helsinki and was approved by the by the regional ethical review board in Stockholm (2021/06932/02) and the Swedish Ethical Review Authority (2020-02826).

## Results

### Prevalence of p.R181H in Sweden and Northern Europe

The population frequency of p.R181H in different cohorts is summarized in Table 1. In the NCI Longitudinal LFS Study comprising 480 patients only eight were found to carry the variant p.R181H (1.7%). A similar proportion of p.R181H carriers is indexed in the NCI *TP53* database of germline *TP53* variant carriers (0.5% of individuals, 0.6% of families). Remarkably, 43 individuals (19 families) in the Swedish *TP53* cohort carried the variant, constituting one quarter (23%) of all individuals and one fifth of all families (22%). Amongst healthy (non-cancer) individuals, the variant was reported in one individual in the FLOSSIES cohort (carrier frequency 0.01%) and four individuals in the non-cancer gnomAD database (carrier frequency 0.001%). Interestingly, the individual in FLOSSIES had European American ancestry and the four individuals in gnomAD all had North European ancestry with two being from Sweden, one from Estonia and one from an unspecified Northwestern European location.

**Table 1:** Proportion of p.R181H carriers in different cohorts.

Cohort	Total patients	p.R181H carriers	Proportion (%)
Swedish cohort (SWEP53)	189	43	23
NCI Longitudinal LFS Study	480	8	1.7
NCI *TP53* database	1,995	10	0.5
FLOSSIES	10,000	1	0.01
gnomAD non-cancer	268,208	4	0.001

### In-silico prediction by AlphaMissense

AlphaMissense was used to computationally predict the pathogenicity of p.R181H. Six different missense variants are possible at this codon; p.R181C, p.R181G, pR181H, p.R181L, p.R181P and p.R181S. AlphaMissense calculates a mean pathogenicity score of 0.821 for these variants with a range of 0.59 to 0.952 (Table S4). Among these variants, the Swedish variant p.R181H is predicted to have the lowest pathogenicity score of 0.59 but is still considered likely pathogenic/pathogenic (cutoff for unknown significance < 0.564).

### Loss of heterozygosity

Tumor DNA was available from two tumors (gastrointestinal stromal tumor and breast cancer, both at age 38 years) in one patient with the p.R181H germline variant in *TP53*. The p.R181H variant was detected in both tumors with high variant allele frequency (VAF) indicative of a loss of heterozygosity of the wild-type *TP53* locus on the short arm of chromosome 17 as a second hit in the tumor (Table S5). This suggests, but does not definitively establish, a pathogenic role of p.R181H in tumorigenesis.

### Distinct phenotypical characteristics

**Fig. 1:**
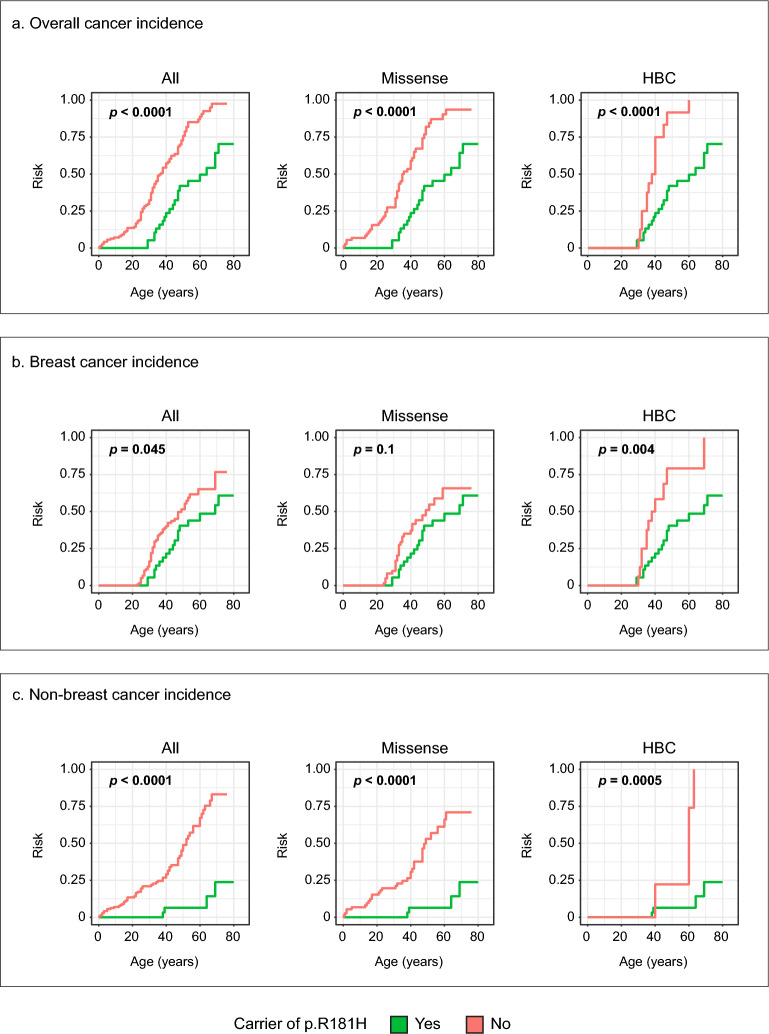
Difference in lifetime cancer risk stratified by p.R181H carrier status in the Swedish cohort. (**a**) Cumulative all cancer incidence for all patients (all), carriers of missense variants (missense) and patients classified as hereditary breast cancer phenotype (HBC). (**b**) Cumulative breast cancer incidence for all patients (all), carriers of missense variants (missense) and patients classified as hereditary breast cancer phenotype (HBC). (**c**) Cumulative non-breast cancer incidence for all patients (all), carriers of missense variants (missense) and patients classified as hereditary breast cancer phenotype (HBC). Green indicates carriers of the *TP53* p.R181H germline variant and red indicates carriers of any other class 4 or 5 *TP53* variant. *P*-values calculated using log-rank test.

The lifetime risk for developing cancer was significantly lower in p.R181H carriers than carriers of other variants (*p* < 0.0001, Fig. 1a). For all comparisons, individuals were assigned exclusively to a single group; p.R181H carriers were excluded from comparator groups such as hereditary breast cancer (HBC) or missense to avoid overlap. The difference in cancer incidence persisted even when only comparing against other missense variants or only comparing against individuals classified with the HBC phenotype (*p* < 0.0001 and *p* < 0.0001, Fig. 1a). Furthermore, p.R181H was associated with a lower breast cancer incidence compared to all other variants as well as other HBC individuals (*p* = 0.045 and *p* = 0.004, Fig. 1b). The risk for all non-breast cancers was also significantly lower in carriers of p.R181H compared to; all other variants (*p* < 0.0001, Fig. 1c), all missense variants (*p* < 0.0001, Fig. 1c) and all patients classified as HBC (*p* = 0.0005, Fig. 1c).

**Fig. 2:**
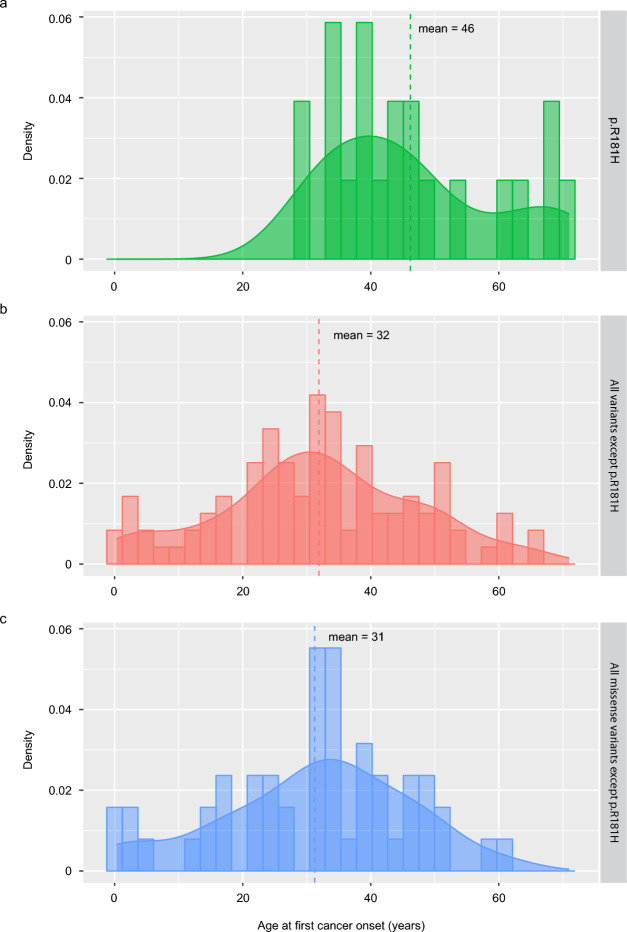
Temporal distribution of age at first cancer onset in the Swedish cohort. (**a**) Carriers of p.R181H. (**b**) Carriers of all variants except p.R181H. (**c**) Carriers of missense variants except p.R181H. Mean age at first cancer onset shown by vertical line with corresponding color (46 years old; 32 years old; 31 years old).

Mean age at first cancer onset was significantly higher in carriers of p.R181H (46 years old) compared to all other variant carriers (32 years old, *p* = 0.0001, Table 2). The density curve for p.R181H carriers is distinctly skewed to the right compared to other variant carriers (Fig. 2). The youngest patient with the p.R181H variant developed a gastrointestinal stromal tumor (her first non-breast malignancy) at the age of 38. (Individual ID 47, concurrent breast cancer, Table S6). In contrast, 33 patients carrying other variants than p.R181H developed non-breast cancers before the age of 38, with the youngest being four months old. Strikingly, none of the p.R181H carriers developed a childhood cancer (< 18 years old) compared to 19% of carriers of other variants (*p* = 0.02, Table 2). Only 7% of p.R181H carriers developed multiple primary cancers as compared to 25% in carriers of other variants (*p* = 0.01, Table 2).

**Table 2:** Clinical characteristics stratified by p.R181H carriership in the Swedish cohort.

Characteristic	Carrier of p.R181H		p-value
	Yes n = 43 (%)	No n = 146 (%)	
Sex			0.3
Male	11 (26)	49 (34)	
Female	32 (74)	96 (66)	
Mean age at variant diagnosis (years)	47	35	0.0007
Mean age at first cancer onset (years)	46	32	0.0001
First cancer onset < 18 years	0	19 (19)	0.02*
Patients with multiple cancers	3 (7)	37 (25)	0.01

Patients with breast cancer	n = 18 (%)	n = 58 (%)	
Mean age at first breast cancer onset (years)	44	36	0.01
First breast cancer onset < 40 years	7 (39)	41 (72)	0.01
Cancer patients with only breast cancer^a^	17 (81)	37 (37)	0.0002*
Patients with multiple breast cancers	1 (6)	13 (22)	0.2*

Mean and independent t-test for continuous variables, chi-squared test for categorical variables. Percentages exclude missing data.

*Fisher’s exact test.

^a^Calculated among all cancer patients in each group.

**Fig. 3:**
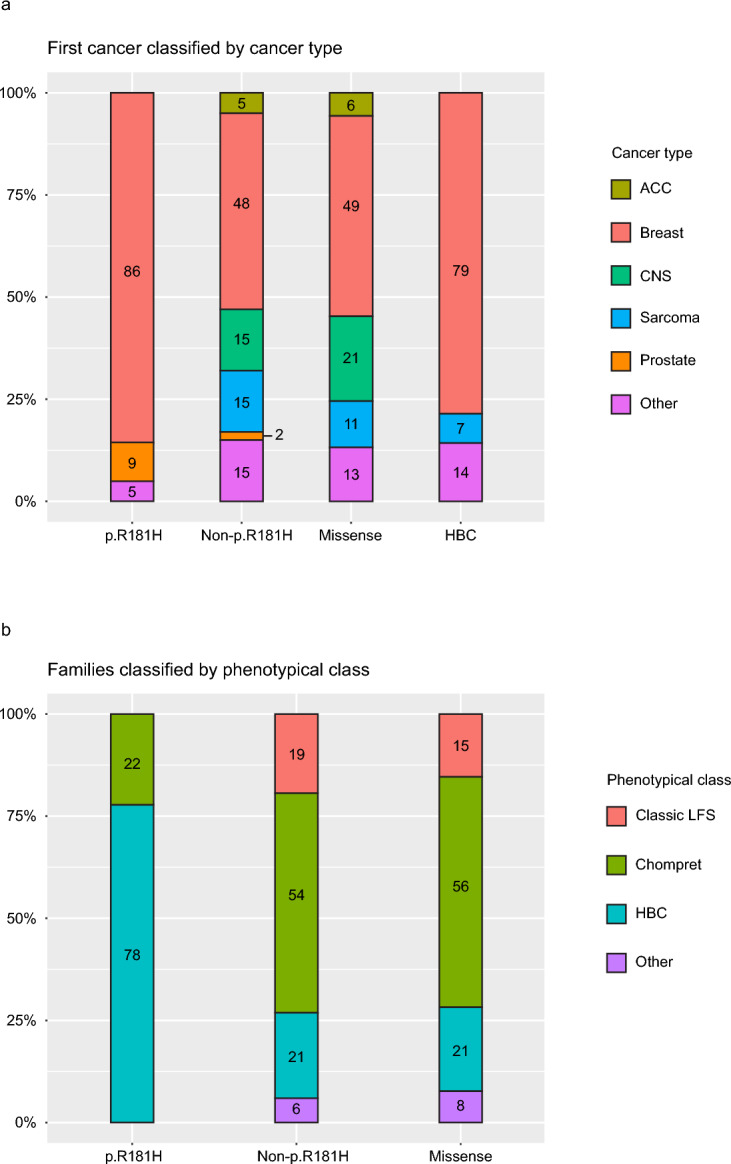
First cancer classified according to core cancers in LFS and distribution of familial phenotypical classification in the Swedish cohort. (**a**) First cancer in each patient classified according to the cancers: adrenocortical carcinoma (ACC), breast cancer, central nervous system (CNS) tumor, sarcoma and prostate cancer, all other cancers classified as “other”. Categorized by only p.R181H carriers (p.R181H), all carriers of other variants (non-p.R181H), missense variants except p.R181H (missense) and patients classified as hereditary breast cancer except p.R181H carriers (HBC). Corresponding proportions written as percentages in each bar. (**b**) Proportion of families classified as the phenotypical classes classic LFS, Chompret and HBC, all other families classified as “other”. Categorized by only p.R181H carriers (p.R181H), all carriers of other variants (non-p.R181H) and missense variants except p.R181H (missense). Corresponding proportions written as percentages in each bar.

Mean age at first breast cancer onset was also higher in p.R181H carriers compared to carriers of other variants (44 years old vs 36 years old; *p* = 0.01, Table 1) and a significantly smaller proportion of breast cancers were diagnosed before the age of 40 (39% vs 72%; *p* = 0.01, Table 1). Furthermore, 81% of cancer patients with p.R181H developed exclusively breast cancers while the corresponding proportion for other variant carriers was 37% (*p* = 0.0002, Table 1). The narrow cancer spectrum is further illustrated in Fig. 3a where breast cancers constitute 86% of all first cancers and 100% of all first cancers in female carriers (Table S6).

All male p.R181H carriers with cancer developed prostate cancer (Fig. 3a), with a mean age at onset of 59 years. No prostate cancers were observed among carriers of other missense variants or in other HBC families. Thus, prostate cancer is enriched in p.R181H carriers compared to all other variants (Fig. 3a).

The most common phenotypical classification for families carrying p.R181H was HBC (78%) while the rest were classified as Chompret. No family was classified as classic LFS (Fig. 3b). In contrast, the HBC phenotype was the least common amongst carriers of other variants (21%, Fig. 3b). The proportion of p.R181H carriers in each phenotypical group is shown in Table S7.

**Fig. 4:**
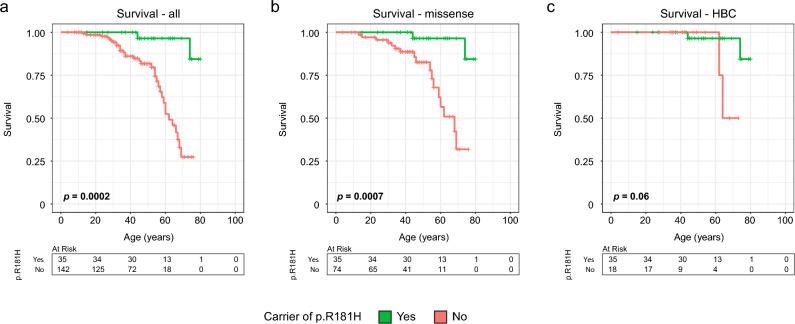
Difference in overall survival stratified by p.R181H carrier status in the Swedish cohort. (**a**) All patients. (**b**) Only carriers of missense variants. (**c**) Only p.R181H carriers and patients with hereditary breast cancer phenotype (HBC). Green indicates carriers of the *TP53* p.R181H germline variant and red indicates carriers of any other class 4 or 5 *TP53* variant. *P*-values calculated using log-rank test.

Overall survival was significantly better amongst the p.R181H group (*p* = 0.0002, Fig. 4a), irrespective of cancer. At 75 years of age, 84% of the patients with p.R181H were still alive as compared to only 27% for other variant carriers (Fig. 4a). The difference in survival persisted even when comparing p.R181H carriers to only carriers of missense variants (*p* = 0.0007, Fig. 4b) but diminished when comparing against individuals with the HBC phenotype (*p* = 0.06, Fig. 4c).

### Confirmation using NCI *TP53* database

**Fig. 5:**
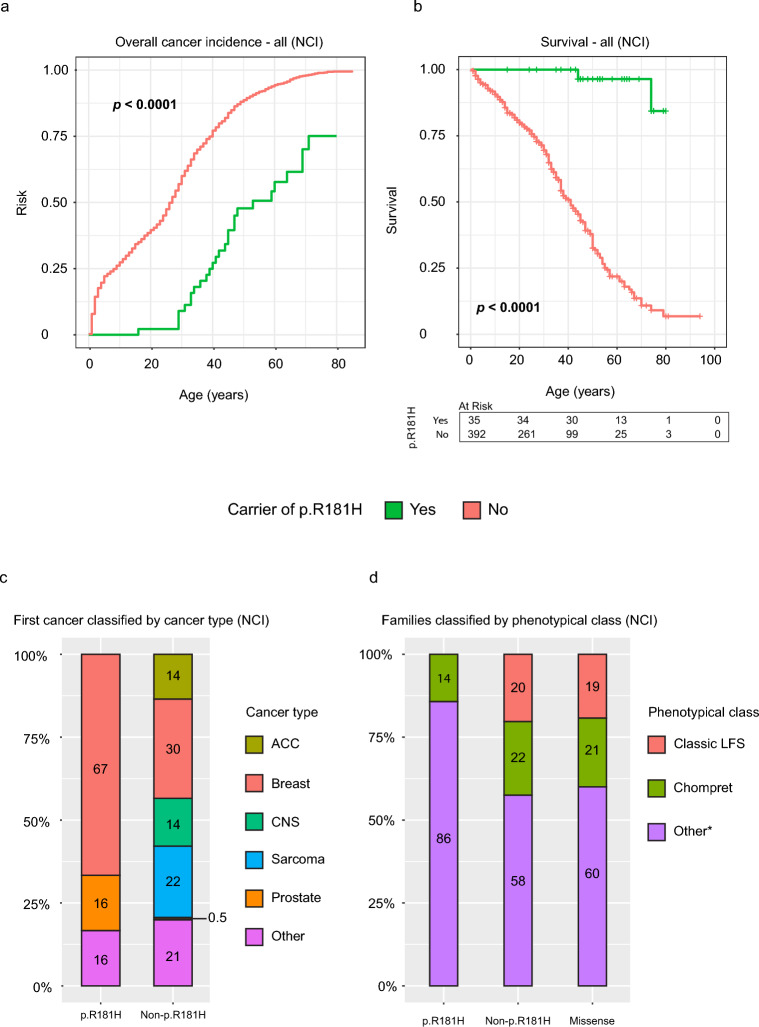
Differences in phenotype between p.R181H and non-p.R181H carriers in the NCI *TP53* database. (**a**) Cumulative cancer incidence comparing both Swedish and NCI *TP53* database p.R181H carriers against all non-p.R181H carriers in the NCI *TP53* database. (**b**) Overall survival comparing Swedish p.R181H carriers against all non-p.R181H carriers in the NCI *TP53* database. No p.R181H carriers in the NCI *TP53* database had survival data. Green indicates carriers of the *TP53* p.R181H germline variant and red indicates carriers of any other class 4 or 5 *TP53* variant. (**c**) Using only the NCI *TP53* database; first cancer in each patient classified according to the cancers: adrenocortical carcinoma (ACC), breast cancer, central nervous system (CNS) tumor, sarcoma and prostate cancer, all other cancers classified as “other”. Categorized by p.R181H carrier status. Corresponding proportions written as percentages in each bar. (**d**) Using only the NCI *TP53* database; proportion of families classified as the phenotypical classes classic LFS, Chompret and HBC, all other families classified as “other”. Categorized by only p.R181H carriers (p.R181H), all carriers of other variants (non-p.R181H) and missense variants except p.R181H (missense). Corresponding proportions written as percentages in each bar. *HBC families are classified as “other” in the NCI *TP53* database.

The clinical characteristics of the p.R181H carriers in the NCI *TP53* database were similar to the 43 Swedish patients and differed from the carriers of other variants in the *TP53* database (Table 3). Carriers of p.R181H had a later age at first cancer onset and only one individual developed a cancer (skin cancer, not LFS core cancer) before the age of 18 (Table 3 and Table S6). Cancer incidence was analyzed by pooling p.R181H carriers from the Swedish and NCI *TP53* cohort and comparing against all other variant carriers in the NCI *TP53* cohort, with significantly lower cancer risk amongst p.R181H carriers (*p* < 0.0001, Fig. 5a). Comparing the p.R181H carriers from the Swedish cohort with other variant carriers in the NCI *TP53* cohort showed significantly better survival amongst p.R181H carriers (*p* < 0.0001, Fig. 5b). Both the cumulative cancer incidence and survival analysis (Fig. 5a,b) are in agreement with the findings based on the Swedish cohort.

**Table 3:** Clinical characteristics stratified by p.R181H carriership in the NCI *TP53* database.

Characteristic	Carrier of p.R181H	
	Yes n = 10 (%)	No n = 1985 (%)
Sex		
Male	3 (30)	607 (33)
Female	7 (70)	1224 (67)
Mean age at first cancer onset (years)	37	25
First cancer onset < 18 years	1 (10)	591 (30)
Patients with multiple cancers	0	531 (27)

Patients with breast cancer	n = 6 (%)	n = 640 (%)
Mean age at first breast cancer onset (years)	37	34
First breast cancer onset < 40 years	2 (50)	400 (75)
Cancer patients with only breast cancer^a^	6 (60)	439 (26)
Patients with multiple breast cancers	0	150 (23)

Percentages exclude missing data.

^a^Calculated among all cancer patients in each group.

Classification of first cancer into core cancer group and phenotypical classification of families was performed exclusively using the NCI *TP53* cohort and showed similar results as in the Swedish cohort. Carriers of p.R181H exhibited a later age of first cancer onset, with a high proportion presenting with breast cancer as their initial or sole diagnosis (Table 3, Fig. 5c). Phenotypical classification of families in the NCI *TP53* cohort showed similar proportions as in the Swedish cohort with no p.R181H families being classified as classic LFS (Fig. 5d).

### HER2 status in breast cancers

The proportion of HER2 positive breast cancers was lower amongst carriers of p.R181H compared to carriers of other variants (33% vs 52%, Table S8), although the difference was not statistically significant (*p* = 0.3).

### Haplotyping

Haplotype analysis was used to evaluate the founder status of the variant and showed two haplotypes flanking a common core shared amongst all families consisting of *TP53* and D17S1353 (Table S3); this could suggest a common genetic ancestor. The time to the most recent common ancestor (TMRCA) was calculated using a computational likelihood-based method (EstiAge) and estimated to be 550 years or 22 generations (95% CI [12, 40]), meaning that the original carrier lived in Sweden during the late 15th or early sixteenth century^27^.

## Discussion

Through our extensive Swedish nationwide characterization of germline *TP53* carriers we identified p.R181H as a unique variant; carriers exhibit a distinct phenotype characterized by lower cancer penetrance, higher proportion of breast and prostate cancers and improved survival compared to carriers of other *TP53* variants.

To date, only three different *TP53* founder variants have been suggested through haplotyping, these are p.R337H (Brazilian), p.G334R (Ashkenazi Jews) and p.R181C (Arabs). All these variants exhibit a wide cancer spectrum including several of the LFS core cancers, with presence of both childhood and adult-onset cancers^16,17,31^. As a result, implications for personalized clinical handling have unfortunately been limited with patients recommended similar surveillance regardless of variant starting from birth^9^.

The newest Swedish guidelines on LFS will recommend no testing or surveillance of pediatric carriers of p.R181H and only breast-MRI and prostate-specific antigen (PSA) testing for adult females and males, respectively^32^. This approach could reduce unnecessary follow-up of non-specific findings from WB-MRI. Approximately one-third of WB-MRI scans in *TP53* carriers lead to false positives and benign follow-ups, often associated with psychological stress and, in some cases, harm from invasive diagnostics^33–36^. Variant-specific recommendations may reduce unnecessary hospital visits and invasive diagnostics, while improving cost-effectiveness by aligning surveillance with actual risk.

A recent literature review and case report suggested p.R181H could be associated with a breast cancer focused phenotype and lower penetrance^37^. However, all previous publications of the variant lack inferential statistics due to very limited sample sizes with the largest cohort published consisting of only ten individuals from four families. Furthermore, no survival data has been presented due to limited follow-up^37,38^. Therefore, there has been no specific guidance for personalized patient management.

By the age of 30 years, only 5% of p.R181H carriers had developed a cancer, which is significantly lower than the 15–20% penetrance observed in carriers of the Brazilian founder variant by the same age^39^. A recent Swedish prospective study sequencing all children with solid tumors from May 2021 to December 2022 found no germline carriers of the p.R181H variant, confirming its low tumor risk in pediatric carriers^40^. The incomplete penetrance of p.R181H was further supported by its presence in the non-cancer cohorts in gnomAD and FLOSSIES.

Furthermore, the variant has a relatively low pathogenicity score, as calculated by AlphaMissense, and shows partially functional transcriptional activity (between 20 and 75% of wild type) in functional studies in yeast^41^. A Danish study used a structure-based protein dynamics approach to investigate the effects of *TP53* variants, including p.R181H. Their results reveal a structural alteration in p.R181H leading to decreased TP53 protein dimer formation, thereby affecting its DNA binding^42^. These structural changes caused by the variant should be further studied to elucidate the potential phenotypical associations.

The haplotype analysis identified two haplotypes with a shared core, excluding the possibility of a hot-spot variant. The small size of the shared core suggests either an ancient founder variant, as indicated by our age estimation software, or the presence of two independent founders. To clarify the variant’s origin, we are initiating collaborations with other European groups to analyze haplotypes in non-Swedish carriers.

In this paper, we present the largest and most comprehensive p.R181H cohort to date. We found its phenotype to be characterized by later-onset breast cancer in females, prostate cancer in males, and no childhood cancers, resulting in significantly improved survival. Our findings suggest that the clinical management of p.R181H carriers could potentially differ from that of carriers of high-risk variants. The data may guide future studies exploring whether management could be optimized by focusing predictive carrier testing and surveillance on adults, and by evaluating an approach that omits whole-body MRI in favor of breast-MRI for adult female carriers and PSA testing for adult male carriers. Thus, the genotype–phenotype correlations of p.R181H enable personalized clinical handling with potential implications for patient care and surveillance recommendations.

## Supplementary Information

Supplementary Information.

## Data Availability

Deidentified data from the Swedish germline *TP53* database is available upon reasonable request by e-mail to the corresponding author. Novel variants have previously been submitted to ClinVar (submission number SUB14867261)^43^.
