# Supplementary material for: TP53 p.R181H is enriched in the Swedish cohort (SWEP53) and associated with a distinct breast and prostate phenotype
Source: Sci Rep. 2025 Oct 7;15:35033. doi: 10.1038/s41598-025-22407-2 (PMC12504530; doi:10.1038/s41598-025-22407-2)
Supplement: Supplementary file 1 — Supplementary Information. [file 41598_2025_22407_MOESM1_ESM.docx]

**Supplemental Table S1.** All *TP53* variants in the Swedish cohort

| **Variant (cDNA)** | **Variant (protein)** | **Effect** | **Number of carriers** |
| --- | --- | --- | --- |
| c.542G>A | p.R181H | missense | 43 |
| c.330del | p.L111Wfs*12 | frameshift | 13 |
| c.733G>A | p.G245S | missense | 12 |
| c.844C>T | p.R282W | missense | 12 |
| c.374C>T | p.T125M | missense | 9 |
| c.993G>A | p.Q331Q | splice^1^ | 9 |
| c.743G>A | p.R248Q | missense | 7 |
| c.919+1G>A | - | splice | 7 |
| c.402T>G | p.F134L | missense | 6 |
| c.455C>T | p.P152L | missense | 6 |
| c.1010G>A | p.R337H | missense | 4 |
| c.1024C>T | p.R342* | nonsense | 4 |
| c.375G>A | p.T125T | splice^2^ | 4 |
| c.586C>T | p.R196* | nonsense | 4 |
| c.715_718dup | p.S240Kfs*25 | frameshift | 4 |
| c.799C>T | p.R267W | missense | 4 |
| c.-29+4088_994-91del | exon 2-9 deletion | large deletion | 3 |
| c.1045G>T | p.E349* | nonsense | 3 |
| c.270del | p.W91Gfs*32 | frameshift | 3 |
| c.473G>A | p.R158H | missense | 3 |
| c.538G>A | p.E180K | missense | 3 |
| c.994-2A>G | - | splice | 3 |
| c.503del | p.H168Pfs*2 | frameshift | 2 |
| c.742C>T | p.R248W | missense | 2 |
| c.140del | p.P47Rfs*76 | frameshift | 1 |
| c.365_366del | p.V122Dfs*25 | frameshift | 1 |
| c.376-1G>T | - | splice | 1 |
| c.395A>G | p.K132R | missense | 1 |
| c.455del | p.P152Rfs*18 | frameshift | 1 |
| c.528C>G | p.C176W | missense | 1 |
| c.636del | p.R213Dfs*34 | frameshift | 1 |
| c.637C>T | p.R213* | nonsense | 1 |
| c.638G>A | p.R213Q | missense | 1 |
| c.659A>G | p.Y220C | missense | 1 |
| c.680del | p.S227Lfs*20 | frameshift | 1 |
| c.708C>G | p.Y236* | nonsense | 1 |
| c.817C>G | p.P273G | missense | 1 |
| c.817C>T | p.R273C | missense | 1 |
| c.818G>A | p.R273H | missense | 1 |
| c.832C>T | p.P278S | missense | 1 |
| c.949C>T | p.Q317* | nonsense | 1 |
| c.96+1G>T | - | splice | 1 |
| - | exon 1 deletion | large deletion | 1 |

^1^Disrupts normal splicing. Exon 9 skipping, leading to a frameshift, premature stop codon and nonsense mediated decay. Shown by our internal data and Magnusson et al. ClinVar variation ID: 428868^24,25^.

^2.^Disrupts normal splicing. Disrupted intron 4 donor site, leading to a frameshift, premature stop codon and nonsense mediated decay. Shown by Catalan Institute of Oncology. ClinVar variation ID: 177825^24^.

**Supplemental Table S2.** Phenotypical criteria used to classify families

| **Classic LFS (n families = 13)** | **Chompret (n families = 40)** | **HBC (n families = 28)** | **Other (n families = 4)** |
| --- | --- | --- | --- |
| • A proband with a sarcoma diagnosed before age 45 years AND   • A first-degree relative with any cancer diagnosed before age 45 years AND   • A first- or second-degree relative with any cancer diagnosed before age 45 years or a sarcoma diagnosed at any age. | • A proband with a tumor belonging to the LFS core tumor (e.g., premenopausal breast cancer, soft-tissue sarcoma, osteosarcoma, central nervous system (CNS) tumor, adrenocortical carcinoma) before age 46 years AND at least one first- or second-degree relative with an LFS tumor (except breast cancer if the proband has breast cancer) before age 56 years or with multiple tumors; OR  • A proband with multiple tumors (except multiple breast tumors), two of which belong to the LFS tumor spectrum and the first of which occurred before age 46 years; OR  • A proband with adrenocortical carcinoma, choroid plexus tumor, or rhabdomyosarcoma of embryonal anaplastic subtype, irrespective of family history; OR  • A female proband with breast cancer before age 31 years. | • Not fulfil the criteria of Classic LFS and Chompret criteria AND  • One case of breast cancer diagnosed under 40 years of age or triple negative breast cancer diagnosed under 60 years of age; OR  • Two cases of breast cancer diagnosed of which at least one under 50 years of age; OR  • Three cases of breast cancer diagnosed of which at least one under 60 years of age; OR  • Breast and ovarian cancer; OR  • Male breast cancer. | •No fulfilment of Classic LFS, Chompret nor HBC criteria |

Classic LFS; classic Li-Fraumeni Syndrome, HBC; hereditary breast cancer. Number of families with each phenotype in parenthesis, one family with unknown classification.

**Supplemental Table S3.** Haplotype analysis using twelve different short tandem repeat (STR) markers in seven different families

| GRCh38 (Mb) | 3.78 | 3.91 | 4.44 | 4.61 | 6.34 | 6.41 | 6.62 | 6.92 | 7.35 | 7.668-7.687 | 7.71 | 8.50 | 8.91 |  |  |
| --- | --- | --- | --- | --- | --- | --- | --- | --- | --- | --- | --- | --- | --- | --- | --- |
| Marker D17S: | 919 | 1828 | 1584 | 675 | 796 | 1149 | 1881 | 578 | 960 | *TP53* | 1353 | 647 | 786 |  |  |
| Family |  |  |  |  |  |  |  |  |  |  |  |  |  |  |  |
| A | | **140** | **209** | **115** | **186** | **142** | **313** | **216** | **148** | **131** |  | **215** | **94** | **144** |  |
| B | **144** | **209** | **113*** | **186*** | **142** | **313** | **216** | **148** | **131** |  | **215** | **94** | **144** |  |  |
| C | 144 | 209 | 113 | 186 | 142 | 313 | 216 | 148 | 131 |  | 215 | 94 | 147 |  |  |
| D | 140/144 | 209/211 | 99/113 | 186 | 142 | 313/316 | 216 | 148 | 131 |  | 215 | 94 | 144 |  |  |
| E | 144/152 | 211 | 99 | 186 | 142 | 313/316 | 219 | 148 | 127 |  | 215 | 74 | 144 |  |  |
| F | | | 152 | 213^#^ | 99 | 186 | 142 | 316 | 219 | 148 | 127 |  | 215 | 74 | 144 |
| G | **144** | **211** | **99** | **186** | **142** | **316** | **219** | **148** | **127** |  | **215** | **74** | **144** |  |  |

Physical position on chromosome 17 and names of markers in title rows. Proven haplotypes through analysis of at least three family members in bold. If two markers are denoted, the results were inconclusive at that locus.

* Two of three individuals had this allele, third carrier did not.

^#^ Alleles overlap in D17S1828

**Supplemental Table S4.** Pathogenicity score of all missense variants at codon 181 computed by AlphaMissense

| **Variant** | | **Pathogenicity score** |
| --- | --- | --- |
| p.R181P | | 0.988 |
| p.R181S | | 0.952 |
| p.R181G | | 0.832 |
| p.R181C | | 0.782 |
| p.R181L | | 0.779 |
| **p.R181H** | | **0.59** |
|  |  |  |

**Supplemental Table S5.** Targeted gene panel sequencing performed on DNA extracted from tumor tissue in a patient who carried the variant in the germline

| **Tumor type** | ***TP53* variant** | **VAF in tumor** | **Second hit in *TP53*** |
| --- | --- | --- | --- |
| Gastrointestinal stromal tumor | p.R181H | 82% | LOH |
| Breast cancer | p.R181H | 68% | LOH |
| LOH; loss of heterozygosity, VAF; variant allele frequency | |  |  |

**Supplemental Table S6.** All p.R181H variant carriers in the Swedish and NCI *TP53* cohorts sorted by family

| **Cohort** | **Family ID** | **Individual ID** | **Sex** | **First cancer (age in years)** | **Second cancer (age in years)** |
| --- | --- | --- | --- | --- | --- |
| Sweden | 1 | 31 | Female | Breast (29) |  |
| Sweden | 1 | 228 | Male | Prostate (64) |  |
| Sweden | 1 | 28 | Male |  |  |
| Sweden | 1 | 29 | Female |  |  |
| Sweden | 1 | 30 | Male |  |  |
| Sweden | 1 | 32 | Female |  |  |
| Sweden | 1 | 50 | Male |  |  |
| Sweden | 5 | 41 | Female | Breast (71) |  |
| Sweden | 5 | 47 | Female | Breast (38) | Gastrointestinal stromal tumor (38) |
| Sweden | 5 | 115 | Male | Prostate (69) |  |
| Sweden | 5 | 116 | Female |  |  |
| Sweden | 5 | 118 | Male |  |  |
| Sweden | 5 | 172 | Male |  |  |
| Sweden | 5 | 42 | Female |  |  |
| Sweden | 5 | 51 | Female |  |  |
| Sweden | 5 | 54 | Male |  |  |
| Sweden | 21 | 86 | Female | Breast (47) |  |
| Sweden | 21 | 98 | Male |  |  |
| Sweden | 28 | 11 | Female | Breast (42) |  |
| Sweden | 28 | 201 | Female | Breast (47) |  |
| Sweden | 28 | 199 | Female |  |  |
| Sweden | 28 | 200 | Female |  |  |
| Sweden | 28 | 221 | Female |  |  |
| Sweden | 205 | 101 | Female | Breast (33) |  |
| Sweden | 206 | 102 | Female | Breast (44) |  |
| Sweden | 207 | 103 | Female | Breast (60) |  |
| Sweden | 208 | 104 | Female | Breast (53) |  |
| Sweden | 209 | 105 | Female | Breast (29) |  |
| Sweden | 210 | 106 | Female |  |  |
| Sweden | 211 | 107 | Female | Breast (34) |  |
| Sweden | 212 | 108 | Female |  |  |
| Sweden | 213 | 109 | Female |  |  |
| Sweden | 214 | 110 | Female | Breast (33) |  |
| Sweden | 215 | 114 | Female | Breast (48) | Breast (54) |
| Sweden | 229 | 61 | Female | Breast (36) |  |
| Sweden | 229 | 135 | Female | Breast (69) |  |
| Sweden | 229 | 137 | Female |  |  |
| Sweden | 229 | 138 | Female |  |  |
| Sweden | 229 | 139 | Male |  |  |
| Sweden | 229 | 140 | Female |  |  |
| Sweden | 240 | 161 | Male | Pheochromocytoma (39) | Prostate (45) |
| Sweden | 257 | 198 | Female | Breast (40) |  |
| Sweden | 274 | 227 | Female | Breast (45) |  |
| NCI | BAE19-1 | 3603 | Male | Thyroid |  |
| NCI | BAE19-1 | 3604 | Male | Skin (16) |  |
| NCI | BAE19-1 | 3605 | Female | Other cancer |  |
| NCI | BorA | 85 | Female | Breast (41) |  |
| NCI | HEY10-6 | 2458 | Female | Breast (29) |  |
| NCI | PAL17-2 | 3658 | Female | Breast (31) |  |
| NCI | PET19-3 | 3813 | Female | Breast (45) |  |
| NCI | SHI17-13 | 3342 | Male | Prostate (59) |  |
| NCI | TUN14-863 | 2883 | Female | Breast |  |
| NCI | ZA01545 | 3372 | Female | Breast |  |

**Supplemental Table S7.** Proportion of the variant p.R181H across phenotypical groups in the Swedish cohort

| **Phenotype** | **Total patients** | **Carrier of p.R181H** | **Proportion (%)** |
| --- | --- | --- | --- |
| Classic LFS | 35 | 0 | 0 |
| Chompret | 105 | 18 | 17 |
| HBC | 42 | 24 | 57 |
| Other | 6 | 0 | 0 |
| Unknown | 1 | 1 | 100 |

**Supplemental Table S8.** Number of patients with HER2 positive and negative breast cancers

| **Carrier of p.R181H** | | **HER2 positive** | **HER2 negative** |
| --- | --- | --- | --- |
| Yes | | 5 | 10 |
| No | | 14 | 13 |
|  |  |  |  |
